# Sensitivity and Responses of Chloroplasts to Heat Stress in Plants

**DOI:** 10.3389/fpls.2020.00375

**Published:** 2020-04-02

**Authors:** Shanshan Hu, Yanfei Ding, Cheng Zhu

**Affiliations:** Key Laboratory of Marine Food Quality and Hazard Controlling Technology of Zhejiang Province, College of Life Sciences, China Jiliang University, Hangzhou, China

**Keywords:** heat stress, chloroplasts, response, photosynthetic, retrograde signals

## Abstract

Increased temperatures caused by global warming threaten agricultural production, as warmer conditions can inhibit plant growth and development or even destroy crops in extreme circumstances. Extensive research over the past several decades has revealed that chloroplasts, the photosynthetic organelles of plants, are highly sensitive to heat stress, which affects a variety of photosynthetic processes including chlorophyll biosynthesis, photochemical reactions, electron transport, and CO_2_ assimilation. Important mechanisms by which plant cells respond to heat stress to protect these photosynthetic organelles have been identified and analyzed. More recent studies have made it clear that chloroplasts play an important role in inducing the expression of nuclear heat-response genes during the heat stress response. In this review, we summarize these important advances in plant-based research and discuss how the sensitivity, responses, and signaling roles of chloroplasts contribute to plant heat sensitivity and tolerance.

## Introduction

Increased temperatures due to climate change pose a severe threat to crop yields worldwide (Wang et al., 2018). Generally, heat stress is often defined as the rise in temperature beyond a threshold level for a period of time sufficient to cause irreversible damage to plant growth and development (Wahid et al., 2007). High temperatures damage the activity of proteins and the fluidity membrane lipids, thus affecting the activity of chloroplast- and mitochondria-based enzymes and membrane integrity. Both severe heat stress and long-term exposure to moderate high temperatures can result in cellular damage and cell death. In tropical and subtropical climates, heat stress may become a major limiting factor in field crop production. Thus, it is imperative that we develop crops with improved heat tolerance by means of heat acclimation of plants, molecular breeding and genetic engineering (Schöffl et al., 1998; Howarth, 2005; Wahid et al., 2007).

During photosynthesis in higher plants, sunlight is trapped and converted into biological energy by chloroplasts. These organelles serve as metabolic centers and play key roles in sensing heat stress and instigating appropriate physiological adaptive responses. Photosynthesis-associated processes including electron transport, CO_2_ assimilation, photophosphorylation, chlorophyll (Chl) biosynthesis, thylakoid membrane fluidity and photochemical reactions are sensitive to heat stress. Normally, these major metabolic processes optimize carbon fixation and growth (Berry and Bjorkman, 1980; Sharkey, 2005; Wahid et al., 2007; Allakhverdiev et al., 2008; Kmiecik et al., 2016; Sun and Guo, 2016). Heat stress-induced damage to chloroplasts leads to the inactivation of heat-sensitive proteins such as Rubisco activase (RCA) and the down-regulation of important chloroplast components, thereby leading to decreased photosynthetic efficiency, redox imbalance and possible cell death (Eamus et al., 1995; Klimov et al., 1997; Salvucci, 2004; Salvucci and Crafts-Brandner, 2004; Wahid et al., 2007; Allakhverdiev et al., 2008; Li et al., 2018). As the photosynthetic apparatus within chloroplasts is prone to damage due to thermal stress, these organelles are key to the activation of cellular heat stress signaling processes (Yu et al., 2012; Sun and Guo, 2016; Chen et al., 2017).

As an essential biological process, protein biosynthesis motivates the growth and development of all living organisms (Shalgi et al., 2013; Li et al., 2018). To respond to heat stress, heat shock proteins (HSPs) have evolved in plants. These proteins act as molecular chaperones to promote the folding and refolding of non-native proteins during protein quality control processes (Vierling, 1991; Baniwal et al., 2007; Kotak et al., 2007; von Koskull-Döring et al., 2007; Schramm et al., 2008). The heat shock transcription factor (HSF), which regulates the expression of HSP genes, recognizes the heat shock cis element (HSE) that is conserved in the HSP gene promoter (Kotak et al., 2007; von Koskull-Döring et al., 2007). A large number of studies have been carried out to investigate the functions of HSFs and HSPs. Although these have greatly increased our knowledge of the heat stress response in plants, our understanding of the regulatory network that controls the heat shock response system is far from complete (Yu et al., 2012).

**FIGURE 1:**
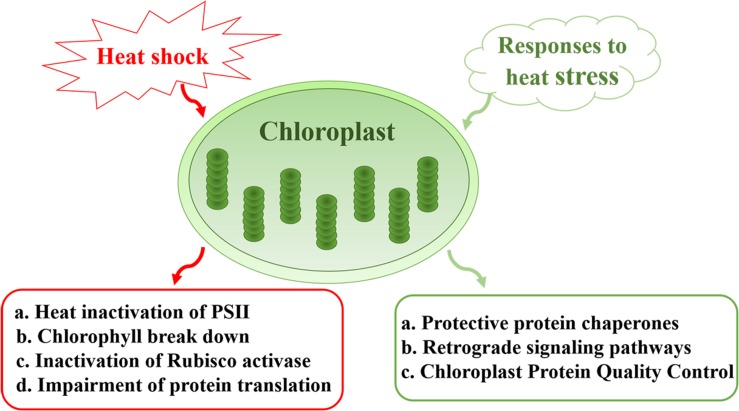
Sensitivity and responses of chloroplasts under heat stress. Major effects of heat stress on chloroplasts include heat inactivation of PSII, Chl breakdown, inactivation of Rubisco, and impairment of protein translation. In response to heat stress, chloroplasts generate a large number of protein chaperones to protect PSII. Meanwhile, chloroplast protein quality control plays a role in maintaining proteostasis under conditions of environmental stress. Chloroplasts also participate in retrograde signal pathways that protect cellular integrity and the normal growth of plants.

In this review, we summarize the impact of heat stress on chloroplast components and the mechanisms that underlie the heat-sensitive nature of this photosynthetic organelle. Heat-induced effects include the inactivation of Photosystem II (PSII), Chl breakdown, inactivation of Rubisco and impairment of protein translation. We also discuss the protective mechanisms that are implemented by chloroplasts in response to heat stress, including the generation of a large number of protein chaperones, and the roles of chloroplasts in retrograde signal pathways that protect cellular integrity and the normal growth of plants under heat stress (Figure 1). The importance and significance of chloroplasts in plant development and productivity under heat stress are also discussed.

## Chloroplast Sensitivity to Heat Stress

### Heat Sensitivity of Photosynthesis

It is well-known that photosynthesis is highly sensitive to high temperatures and heat damage leads to cellular energy imbalance. This is mainly reflected in the distinct alteration to the redox state related to the injury of thylakoid membranes (Berry and Bjorkman, 1980; Biswal et al., 2011). At high temperatures, the photochemical reactions in the thylakoid lamellae and carbon metabolism in the stroma of chloroplasts are most affected (Wise et al., 2004; Wang et al., 2018). Heat stress could directly damage the photosynthetic apparatus, such as PSI and PSII, the cytochrome b6f (Cytb6f) complex and Rubisco (Havaux, 1993a, b; Wise et al., 2004); moreover, heat-induced inactivation of these photosynthetic components can lead to the inhibition of various redox and metabolic reactions (Mathur et al., 2014).

### Photosystem II

Of the chloroplast thylakoid membrane protein complexes, PSII is the most sensitive target of heat stress. Photosynthetic electron transport and ATP synthesis are greatly affected if PSII suffers from severe thermal damage (Wang et al., 2018). Under high temperatures, due to the increased fluidity of thylakoid membranes, PSII light-harvesting complexes fall off the thylakoid membranes, which results in impaired PSII integrity and affects photosynthetic electron transfer (Baker and Rosenqvist, 2004; Janka et al., 2013; Mathur et al., 2014). The oxygen evolving complex (OEC) in PSII is dissociated by heat stress, which further results in the inhibition of electron transport from the OEC to the acceptor side of PSII (Havaux and Tardy, 1996; Allakhverdiev et al., 1997; Wahid et al., 2007; Allakhverdiev et al., 2008).

High temperature stress is accompanied by oxidative stress in plants. A recent study revealed that reactive singlet oxygen, ^1^O_2_, is produced as a byproduct of photosynthesis and can damage PSII reaction center proteins, which in turn induces the PSII repair cycle (Dogra and Kim, 2019). It is also well known that PSII is extremely susceptible to light damage (Aro et al., 1993; Tikkanen et al., 2012). Recent reports suggest that *AhVDE* [a peanut (*Arachis hypogaea* L.) violaxanthin de-epoxidase gene] can mitigate PSII photoinhibition induced by heat and high irradiance stress (Yang et al., 2015). Xanthophyll cycle-dependent non-photochemical quenching (NPQ) can protect PSII under excess irradiance by converting surplus light energy into heat energy to alleviate the excitation energy pressure on PSII reaction centers (Morosinotto et al., 2002; Yang et al., 2015). Previous studies suggest that Zeaxanthin plays an important role in the NPQ mechanism (Niyogi et al., 1998; Nilkens et al., 2010). As is known to all, PSII splits water by absorbed light energy and then transmits electron for the next reaction. However, PSII is susceptible to environmental stress, especially heat stress and oxidative stress. These stresses might inhibit the repair of the photodamaged PSII by inhibition of PSII protein synthesis (Takahashi and Murata, 2008), thus leading to the decrease of photosynthetic efficiency in plants.

### Chlorophyll

**FIGURE 2:**
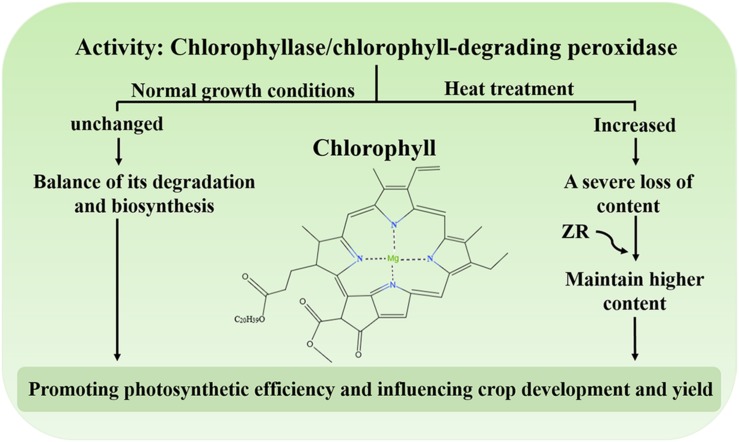
The activity of chlorophyllase and Chl-degrading peroxidase under heat stress. Under normal growth conditions, biosynthesis and degradation of Chl are maintained at steady rates. Under heat stress, the activity of chlorophyllase and chlorophyll-degrading peroxidase increases and the content of Chl is severely reduced. Exogenous application of ZR maintains a higher content of Chl.

Chl, the main photosynthetic pigment in the thylakoid membrane of chloroplasts, could harvest light energy and drive electron transfer during the initial and indispensable processes of photosynthesis (Wang et al., 2018). Studies have shown that degradation of Chl could protect plant cells from the hazardous effects of phototoxic pigments (Ginsburg et al., 1994; Hörtensteiner, 2006, 2009). Under normal growth conditions, the synthesis and degradation of Chl reach equilibrium and the levels of this molecule remain stable (Ginsburg et al., 1994; Hörtensteiner, 2006; Wang et al., 2018). Conversely, when plants are subject to environmental stress, including heat, the content of Chl decreases, leading to leaf senescence or chlorosis (Kim and Nam, 2007; Lim et al., 2007; Allakhverdiev et al., 2008; Rossi et al., 2017). Under heat treatment, the activity of chlorophyllase and Chl-degrading peroxidase dramatically increases, resulting in a serious reduction in Chl levels (Figure 2; Wang et al., 2018). One study demonstrated that the exogenous application of zeatin riboside (ZR) increases Chl levels by reducing the activity of protease in creeping bentgrass during heat stress (Figure 2; Veerasamy et al., 2007). Moreover, degradation of Chl might also be a protective response of plants. Excessive light energy captured by chlorophyll could also threaten the photosynthesis in plants. The study has shown that an ABA biosynthesis-defective mutant *nced3nced5* had less Chl content compared with the wild type under high light (Huang et al., 2019). It may be shows that ABA could regulates Chl biosynthesis under high light. The balance of Chl biosynthesis and breakdown is crucial to maintain the photosynthetic apparatus to promote photosynthetic efficiency, ultimately influencing crop development and yield.

### Ribulose-1, 5-Bisphosphate Carboxylase/Oxygenase (Rubisco)

Rubisco is not only an important carboxylase in C3 carbon reaction of photosynthesis, but also an indispensable oxygenase in photorespiration. During the photosynthetic process, the enzyme Rubisco is responsible for fixing an estimated 1,011 tons of atmospheric CO_2_ (Field et al., 1998; Galmés et al., 2013). Within the Calvin–Benson–Bassham cycle, Rubisco catalyzes the carboxylation of the 5-carbon sugar ribulose-1, 5-bisphosphate (RuBP) (Wang et al., 2018). Under moderately elevated temperatures, the activity of RCA is inhibited, resulting in the thermal inactivation of Rubisco activity (Spreitzer and Salvucci, 2002; Portis, 2003; Sharkey, 2005; Sage et al., 2008; Perdomo et al., 2017). Under high temperatures, the stability of Rubisco’s chaperone activating enzyme decreases, which results in the inhibition of photosynthesis in plants. These results support the theory that RCA is a limiting factor in photosynthesis when the atmospheric temperature exceeds the optimum range for plants (Wang et al., 2018). Studies have shown that photosynthesis is not completely limited by carbon and probably caused by stomata closure. Stomata closure could prevent excessive water loss and increases the temperature of the leaves, thereby increasing the absorption of heat and the photorespiration of Rubisco (Losciale et al., 2014). Moreover, as temperature rises, the affinity of Rubisco for CO_2_ decreases, which tends to catalyze its oxygenase reaction. It would result in the increase of photorespiration and reduce the efficiency of photosynthesis, ultimately leading to reduced crop yields. Recent studies found that a tomato (*Solanum lycopersicum*) chloroplast-targeted DnaJ protein (SlCDJ2) could maintain of CO_2_ assimilation capacity mainly by protecting Rubisco activity under heat stress (Wang et al., 2015). SDJ20 might enhance the carboxylase activity of Rubisco under high temperature stress. Large-scale replacement of the amino acid sequence in the active center of Rubisco enzyme and reprogramming of the coding genes may effectively reduce or remove the damage caused by photorespiration to plants, but it is still difficult to achieve by current biotechnology methods.

### Heat Sensitivity of Chloroplast Protein Translation

As a semi-autonomous organelle, the chloroplast has an independent genome that is used mainly to encode ribosomal proteins and protein molecules that are closely related to photosynthesis. However, heat stress usually leads to the aggregation and denaturation of proteins. The downregulation of nuclear photosynthetic genes in higher plants is caused generally by the inhibition of plastid translation (Beck, 2005; Nott et al., 2006; Bräutigam et al., 2007). Translation elongation factor Tu (EF-Tu) is a conserved GTP-binding protein that is essential to the translation of proteins in prokaryotes and eukaryotic mitochondria and plastids (Li et al., 2018). Li et al. (2018) found that the *Arabidopsis* plastid EF-Tu, *Rabe1b*, is associated with plastid translation under heat stress. Knockdown mutants of *Arabidopsis rabe1b*, virus-induced silencing of tomato *Rabe1b* or overexpression of constitutive GTP- or GDP-bound mutant forms of *Rabe1b* in *Arabidopsis* all reduce heat tolerance. These results indicate that heat-sensitive plastid EF-Tu is associated with the plant response to heat stress (Li et al., 2018). In addition, Yu et al. (2012) confirmed that a retrograde pathway is involved in the regulation of heat stress. They found that chloroplast ribosomal protein S1 (RPS1) is a heat-responsive protein. Importantly, in both *rps1* and *Rabe1b* mutants, the induction of nuclear heat-responsive genes regulated by the HSF HsfA2 is also compromised. These results indicated that chloroplast protein translation is required to induce retrograde signaling in the nuclear heat stress response (Yu et al., 2012; Yang and Guo, 2014). Therefore, chloroplast protein translation functions are important not only in the synthesis of chloroplast proteins that are required for normal function, but also in the communication between plastids and nuclear and cytoplasmic compartments of plant cells.

## Protective Responses of Chloroplasts to Heat Stress

### Protective Role of Chloroplast Protein Chaperones

If chloroplasts are damaged during their development, photosynthetic efficiency is decreased, which leads to a severe reduction in plant productivity (Dutta et al., 2009). However, high temperatures induce the expression of protective chaperones and modulate growth responses (Dickinson et al., 2018). It is well-known that HSPs including Hsp101, Hsp100, and small HSPs (sHsp) play essential roles in responding to heat stress. HSPs, as molecular chaperones, can prevent protein denaturation and aggregation (Barua et al., 2003). Genetic analysis has shown that HSP101 is essential to plant thermotolerance and survival under high-temperature stress (Hong and Vierling, 2001; Myouga et al., 2006; Mathur et al., 2014). *Arabidopsis hsp101* mutants were found to be sensitive to high temperature (Hong and Vierling, 2000), while *Hsp101* overexpressing plants displayed increased tolerance to thermal stress (Queitsch et al., 2000). Myouga et al. (2006) used albino or pale-green (*apg*) mutants containing a *Ds* insertion in the gene encoding APG6 (ClpB3) to reveal it as a homologue of Hsp101. The study demonstrated the roles of nuclear genes in chloroplast development and pigment synthesis. In *apg6* mutants, chloroplast proteins related to photosynthesis were markedly decreased. Further analysis suggest that, as a molecular chaperone, APG6 mediates internal thylakoid membrane formation during plastid differentiation and confers thermotolerance to chloroplasts under heat stress (Myouga et al., 2006).

Hsp100 proteins are molecular chaperones involved in many important metabolic processes of prokaryotes and eukaryotes (Schirmer et al., 1996). Accumulating data indicate that HSP100 is also vital to heat tolerance. Yang et al. (2006) isolated the *Lehsp100/ClpB* gene for HSP100 from tomato chloroplasts. The expression of *Lehsp100/ClpB* was inhibited in antisense transgenic tomatoes, leading to marked impairment of their photosynthetic function. LeHSP100/ClpB proteins are thought to protect PSII against heat stress (Yang et al., 2006). In addition, small HSPs in chloroplasts are associated with the thylakoids and protect PSII under heat and other stresses, possibly by stabilizing the OEC (Heckathorn et al., 2002). Most small HSPs are highly expressed under heat stress and improve heat resistance by protecting proteins from irreversible denaturation (Sun et al., 2002; Sun and MacRae, 2005; Zhong et al., 2013). For example, HSP21 is a nuclear-encoded chloroplast-localized small HSP. It plays an essential role in protecting PSII against heat stress (Heckathorn et al., 1998; Härndahl et al., 1999; Wang and Luthe, 2003; Shakeel et al., 2011; Kim et al., 2012; Zhong et al., 2013). Studies have found that the loss of HSP21 function severely affects seedling development and this small HSP interacts with plastid nucleoid protein pTAC5 to maintain the function of plastid-encoded RNA polymerase (PEP) and plays an essential role in chloroplast development (Zhong et al., 2013). Moreover, Hu et al. (2015) revealed that sHSP26 improved maize chloroplast performance under heat stress by interacting with specific proteins (Hu et al., 2015).

The Orange (Or) protein functions as a holdase chaperone and regulates carotenoid biosynthesis and environmental stress in plants (Zhou et al., 2015; Park et al., 2016; Kang et al., 2017). After heat shock, the efficiency of PSII and chlorophyll contents are improved by the overexpression of the *Or* gene (*IbOr*) from *Ipomoea batatas* (L.) Lam in transgenic *Arabidopsis* (Park et al., 2016; Kang et al., 2017). In higher plants, oxygen-evolving enhancer protein 2-1 (PsbP) protein is necessary for normal thylakoid structure. PsbP can also regulate and stabilize the PSII (Yi et al., 2007, 2009). Sweet potato plants can acquire heat tolerance by stabilizing IbPsbP using the holdase chaperone function of IbOr and stabilizing PSII (Kang et al., 2017). Collectively, chloroplast HSPs and other protein chaperones play important roles in acquiring thermtolerance and protecting chloroplast development and photosynthesis under heat stress.

### Chloroplast Protein Quality Control

Majority of proteins in chloroplasts are encoded by nuclear genes and synthesized in the cytoplasm, and only a small part of the proteins are synthesized by themselves. TOC (transocon at the outer membrane of chloroplasts) and TIC (transocon at the inner membrane of chloroplasts) complexes at the double membrane envelope of chloroplasts mediate the import of the majority of nucleus-encoded proteins into plastids. Extensive studies have shown that the plastid protein import machinery is an important organellar protein targeting system (Abdallah et al., 2000; Leister, 2003; Li and Chiu, 2010; Shi and Theg, 2013; Paila et al., 2015; Richardson et al., 2017; Sjuts et al., 2017). The normal function of chloroplasts depends on the assembly and homeostasis of a large number of nucleus-encoded proteins (Ling et al., 2019). Therefore, Chloroplast protein quality control is important for plans to response to the environmental stress, including heat stress. Upon environmental stimulation, damaged proteins in chloroplasts would be rapidly degraded to ensure normal photosynthesis. Under extreme stress, even the entire chloroplast must be removed to ensure normal plant growth.

The thermal stability of protein import apparatus is quite low. After intact chloroplasts were exposed to 40°C, the protein import inhibition rate reached 100%, but in intact plants, exposed to high temperatures of 40°C for up to 24–48 h, protein introduction is only partially reduced 49–67% (Dutta et al., 2009). The TOC complexes control the initial recognition and translocation of precursor proteins at the outer membrane. Toc159 and Toc34 share a highly conserved GTP-binding domain that initially recognize the chloroplast targeting peptide (Kouranov and Schnell, 1997; Keegstra and Froehlich, 1999; Chen et al., 2000; Sveshnikova et al., 2000; Kessler and Schnell, 2002, 2006; Becker et al., 2004; Dutta et al., 2009). The polypeptide transport-associated (POTRA) domains of Toc75 orient toward the intermembrane space and prevent preproteins from misfolding there (Chen et al., 2016; Paila et al., 2016; Richardson et al., 2017). Moreover, Tic22 is a chaperone in the intermembrane space and facilitates the continuity of precursor translocation between the TOC and TIC channels (Richardson et al., 2017). Tic20 is an essential component of protein import apparatus (Chen et al., 2002; Teng et al., 2006). The outer-membrane channel Toc75 can bind directly to Tic236, which is anchored to the inner membrane (Chen et al., 2018). Dutta et al. (2009) found that the impairment of protein import in heat-stressed plants was mainly due to a decrease in preprotein binding resulting from a decrease in *Toc159* expression. The inhibition of import efficiency may reach 67%, which may be due to the down-regulation of gene/protein expression of certain components of the TOC complex (Toc75), the TIC complex (Tic20, Tic32, Tic55, and Tic62), stromal Hsp93 and stromal processing peptidase (Dutta et al., 2009). In addition, Shakeel et al. (2013) performed a qualitative and quantitative proteomic analysis of the chloroplast stromal proteome. They found that ∼25% of the proteins were membrane proteins in both heat-treated and control samples, only 1–7% of the proteins were found to be involved in functions related to the cytoskeleton, mitochondria, ribosome, and cytoplasm. Therefore, these proteins could be grouped as transport-related or defense-related proteins (Shakeel et al., 2013).

When plants are exposed to high temperatures, the chloroplasts are unfolded and vulnerable to proteolytic enzymes, resulting in rapid degradation of chloroplast proteins (Dutta et al., 2009). It is generally known that the D1 protein, one of the major core subunits in PSII, is the main site susceptible to damage by heat stress or high light (Murata et al., 2007; Yamamoto et al., 2008; Su et al., 2014). The hetero-hexameric FtsH protease which is located in the thylakoid membrane, is essential in degrading damaged PSII reaction center proteins. Previous study found that an FtsH protease(s) was involved in the primary cleavage of the D1 protein under moderate heat stress (Yoshioka et al., 2006). Moreover, the study found that the loss of the central subunit of the FtsH protease, FtsH2, weakened the PSII repair (impaired by heat stress), thereby impairing PSII proteostasis (Dogra and Kim, 2019). After the damaged protein is degraded, a newly synthesized protein is required to replace it to perform normal functions. Echevarría-Zomeño et al. (2016) found two chloroplast ribosomal proteins proteins, PSRP3 and PRPL28 by proteomic analysis (Romani et al., 2012; Tiller et al., 2011). The levels of PSRP3 and PRPL28P increased during committed to survival treatment (38°C for 1 h, set back to 22°C for 1 h and then followed by a challenge of 45°C during 3 h and set back to 22°C for 5 h) (Echevarría-Zomeño et al., 2016). It is reasonable to speculate that PSRP3 and PRPL28P might be related to chloroplast protein synthesis. Therefore, it is essential for plants to maintain proteostasis in chloroplasts under environmental stress. Furthermore, enhanced PSII proteostasis and the accumulation of damaged proteins may facilitate salicylic acid synthesis through the isochorismate pathway established by chloroplasts. Salicylic acid may act as a retrograde signaling molecule for the regulation of nuclear gene expression (Dogra and Kim, 2019). Therefore, there is also a correlation between chloroplast protein homeostasis and retrograde signaling pathways.

### Chloroplast-Associated Retrograde Signaling in Plant Heat Stress Responses

The chloroplast is an environmental sensor that affects the expression of nuclear genes through retrograde signaling. Therefore, it is not surprising that these organelles play an important role in activating cellular heat stress signals (Chan et al., 2016; Dickinson et al., 2018; Wang et al., 2018). Nuclear gene expression is regulated by nuclear-encoded proteins targeted to chloroplasts and retrograde signals from plastids to the nucleus (Rintamäki et al., 2009). Under high temperatures, the heat shock response triggers increased transcription of *HSF* and *HSP* genes (Yu et al., 2012). Chloroplasts transmit environmental stimuli to the nucleus through retrograde signaling pathways by utilizing reactive oxygen species (ROS), tocopherols and chlorophyll biosynthetic intermediates to regulate gene expression in the nucleus.

### HsfA2-Dependent Retrograde Signaling

Protection against high temperature stress in eukaryotes is coordinated by HSFs, transcription factors that activate the expression of protective chaperones. HSFs regulate the expression of *HSP* genes at the transcriptional level by recognizing HSEs that are conserved in *HSP* gene promoters (Kotak et al., 2007; von Koskull-Döring et al., 2007). HsfA2 is a major HSF in the heat-induced expression of nuclear heat-responsive genes in plants and a key regulator of heat tolerance in plants including tomato (Scharf et al., 1998; Döring et al., 2000; Chan-Schaminet et al., 2009) and *Arabidopsis* (Nishizawa et al., 2006; Schramm et al., 2006; Charng et al., 2007). In *Arabidopsis*, *Hsf1*, *Hsf3*, *HsfA2*, and *HsfA3* are all associated with heat tolerance but *AtHsfA2* is most strongly induced by a rise in temperature (Ogawa et al., 2007; Zhang et al., 2018). In tomato, HsfA2 is highly stable and under heat shock conditions translocates into the nucleus upon binding to HsfA1 to form a hetero-oligomer (Scharf et al., 1998; Heerklotz et al., 2001). Interestingly, AtHsfA2 from *Arabidopsis* can also enter the nucleus from the cytoplasm and activates the expression of a series of *Hsp* or chaperone genes in the absence of AtHsfA1 (Schramm et al., 2006).

**FIGURE 3:**
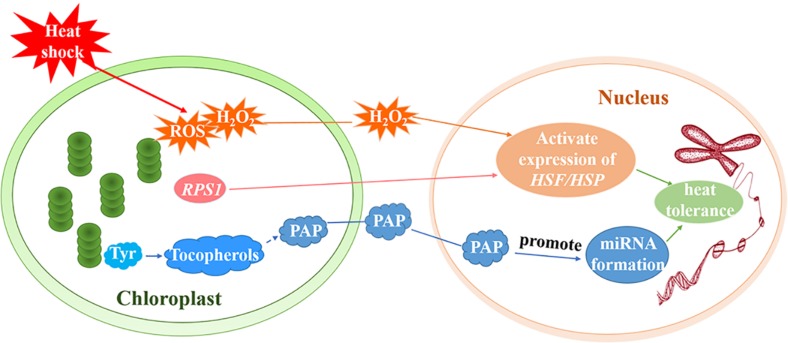
Retrograde signal pathways in chloroplasts under heat stress. Under heat treatment, ROS including H_2_O_2_ as a signaling molecule, induce the activation of HSEs and subsequently increase the expression of HSPs. *RPS1* may activate the expression of the *HsfA2*-dependent heat-responsive gene in the nucleus. Tyr regulates the production of miRNAs by producing downstream metabolite tocopherols (vitamin E). The generation of tocopherols and PAP prevents the degradation of primary miRNAs and promotes the formation of miRNAs to increase the heat tolerance of plants.

ROF1 (AtFKBP62) interacts with HSP90.1 and regulates the HSF, HsfA2, to induce heat tolerance. Meiri and Breiman (2009) reported that the HSP90.1-ROF1 complex appeared in the nucleus and cytoplasm under heat treatment. It is possible that heat stress activates HsfA2 and other unknown factors, leading to the nuclear translocation of the ROF1-HSP90.1 complex (Meiri and Breiman, 2009). RPS1 is responsive to heat stress and also participates in retrograde activation of heat stress responses in higher plants. *Arabidopsis rps1* mutant plants are impaired in the activation of *HsfA2*-dependent heat-responsive gene expression in the nucleus (Figure 3), which is necessary for thermotolerance in higher plants (Yu et al., 2012). In addition, down-regulation of *RPS1* can inhibit thylakoid membrane-associated physiological processes. It affects the generation of ROS such as H_2_O_2_ under heat stress and influences the activation of ROS-mediated retrograde signal transduction (Yu et al., 2012).

### ROS as Retrograde Signals

The aerobic metabolism of plants unavoidably generates ROS such as hydrogen peroxide (H_2_O_2_), superoxide, hydroxyl radicals (⋅OH), and singlet oxygen (^1^O_2_) (Sun and Guo, 2016). ROS serve as a plastid signals and instruct the nucleus to activate the expression of genes that encode antioxidant enzymes and adjust the stress-response mechanisms. It is believed that, under conditions of stress, ROS trigger various operational retrograde signaling pathways and play vital roles in the acclimation of plants (Pogson et al., 2008; Galvez-Valdivieso and Mullineaux, 2010; Suzuki et al., 2012; Sun and Guo, 2016; Wang et al., 2018). Yao et al. (2017) demonstrated that ROS are important in the early heat shock response and may function as major signaling molecules (Yao et al., 2017). H_2_O_2_, for example, is a signaling molecule that activates core transcription regulators under heat stress. Volkov et al. (2006) determined that H_2_O_2_ activates HSFs (Figure 3; Volkov et al., 2006) and subsequently induces *HSP70* expression. Thus, H_2_O_2_ triggers the transduction of a signal to the nucleus that induces the activation of heat-associated gene expression (Dickinson et al., 2018). In fact, the singlet oxygen pathway, which is independent of Mg-protoporphyrin IX (Mg-ProtoIX), is the most intensely studied ROS-dependent retrograde signaling pathway in higher plants (Suzuki et al., 2012). One study revealed that ^1^O_2_ can oxidize β-carotene to produce β-cyclocitral (Ramel et al., 2012; García-Plazaola et al., 2017) but the molecules that transmit the plastid ^1^O_2_ signal out of the chloroplast remain to be identified (Ramel et al., 2013).

### Tocopherols as a Retrograde Signal

The regulation of transcription by retrograde signals is at the center of nuclear gene expression control. MicroRNAs (miRNAs) are dynamically and delicately regulated by transcriptional and post-transcriptional mechanisms. These mechanisms influence the processing and stability of primary and/or mature miRNAs (Rogers and Chen, 2013; Ha and Kim, 2014; Fang et al., 2018). Fang et al. (2018) found that miRNA biogenesis could be regulated by tocopherols (vitamin E). Phosphoenolpyruvate (PEP) is mainly metabolized to produce aromatic amino acids (Tyr, Phe, and Trp) via the shikimate pathway (Fischer et al., 1997; Streatfield et al., 1999; Fang et al., 2018). However, genetic analysis has revealed that Tyr, not Phe or Trp, is essential to the accumulation of miRNAs. Tyr regulates the production of miRNAs by producing downstream metabolite tocopherols. Tocopherols could protect primary miRNAs from degradation and nuclear RNA exonuclease (XRN) negatively controls primary miRNA levels (Fang et al., 2018). Estavillo et al. (2011) found that 3′-phosphoadenosine 5′-phosphate (PAP) is the chloroplast retrograde inhibitor of XRNs (Estavillo et al., 2011). Therefore, tocopherols could inhibit the activity of XRN through chloroplast retrograde signaling molecule PAP, preventing the degradation of primary miRNAs and promoting the formation of miRNAs (Figure 3). Interestingly, the induction of PAP under heat seems to depend on the induction of tocopherols. The study has been shown that primary miR398 and mature miR398 accumulate downstream of PAP induction. Under heat stress, miR398 is induced, and its target gene COPPER/ZINC SUPEROXIDE DISMUTASE2 (CSD2) is downregulated to protect plants against high temperatures. Heat induces the production of tocopherols and PAP, which is required to increase miR398 accumulation and improve thermotolerance (Fang et al., 2018).

### Plant Hormones as Signals

In addition to the above-mentioned chloroplast-associated retrograde signals, the biosynthesis and signal transduction of some plant hormones introduced by some transcriptomic studies may also affect the photosynthesis of chloroplast. Interestingly, after excluding the effects of heat stress on plants, Huang et al. (2019) found that an abscisic acid (ABA) biosynthesis-defective mutant *nced3nced5* was hypersensitive to high light and had less chlorophyll contents compared with the wild type under high light. Plants could dynamically regulate hormones (particularly ABA), photosynthesis, Blue/UV-A photoreceptors, etc. to response to high light (Huang et al., 2019). It can be seen that high light would also affect the photosynthesis of plants, even more damage to plants than high temperature. The biosynthesis of jasmonic acid (JA) involves the translocation of lipid intermediates from the chloroplast membranes to the cytoplasm and then into peroxisomes (León, 2013). Balfagón et al. (2019) revealed that JA is required for regulating several transcriptional responses unique to the stress combination (heat stress and high light). They also found that chloroplast structures and expression of transcripts that encode proteins involved in the photosynthesis changed under high light, heat stress, and combination of high light and heat stress, especially the levels of D1 protein in PSII. Moreover, compared to CT, the quantum yield of PSII and maximal efficiency of PSII (Fv/Fm) values of JA-deficient mutant *aos* markedly decreased under combination of high light and heat stress (Balfagón et al., 2019). Therefore, JA may act as a signal molecule to regulate plant photosynthesis under combination of high light and heat stress, but the signal pathway needs further experiments to explore and verify.

## Conclusion and Future Prospects

In many parts of the world, high temperature has become one of the most important restrictions to plant growth and development, particularly influencing the photosynthetic capacity of plants. Extensive studies on the responses and adaptation of crops to high temperatures have expanded our understanding of thermal stress responses in these organisms. In this review, we have discussed the roles of chloroplasts in heat stress responses from an organellar perspective. High temperatures negatively affect many photosynthetic processes in chloroplasts including chlorophyll biosynthesis, photochemical reactions, electron transport and CO_2_ assimilation. Heat stress leads to the impairment of plastid-based protein translation and under high temperatures, newly synthesized proteins could misfold and existing proteins may be denatured (Ellis, 1990; Bita and Gerats, 2013). Chloroplast heat stress responses are therefore essential to reducing damage and increasing survival rates for plants under high temperatures.

As sessile organisms, plants, especially agricultural crops, are sensitive to elevated temperatures. Under heat stress, various genes and proteins are induced and regulated to protect the normal function of chloroplast and improve the heat tolerance of plants. Plants have evolved complex signaling pathways to sense and respond to heat stress. The interrelation between chloroplasts and the nucleus, termed retrograde signaling, under heat stress has received much attention in recent years. However, the mechanisms and signaling pathways involved in the development of thermotolerance in plants are far from completely understood. We can analyze transcriptomics and proteomics data to screen genes and proteins related to chloroplast heat resistance in order to better understand plant heat resistance mechanisms and signaling pathways. Furthermore, a better understanding of the chloroplast response to heat stress is valuable to the development of new strategies directed at improving crop yield under thermal stress. We could also conduct thermotolerance breeding through genetic engineering and molecular biological techniques to pave the way for providing high-yield economic crops.

## Author Contributions

SH and YD conceived and designed content framework of the review. CZ proposed valuable opinions and ideas. CZ and YD revised the review. SH wrote the manuscript. All authors red and approved the manuscript.

## Conflict of Interest

The authors declare that the research was conducted in the absence of any commercial or financial relationships that could be construed as a potential conflict of interest.
